# Supplementary figures and images for: Langerhans cells drive Tfh and B cell responses independent of canonical cytokine signals
Source: Front Immunol. 2025 Jul 18;16:1611812. doi: 10.3389/fimmu.2025.1611812 (PMC12315773; doi:10.3389/fimmu.2025.1611812)

Fig. S1

A

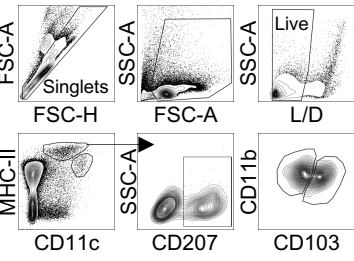

B

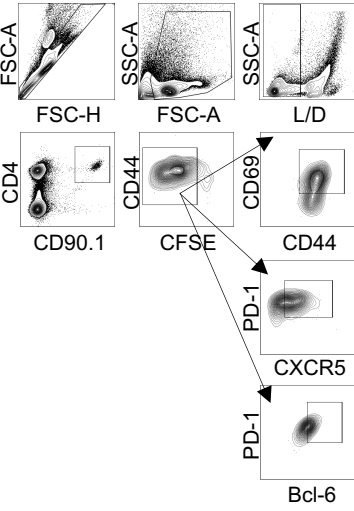

C

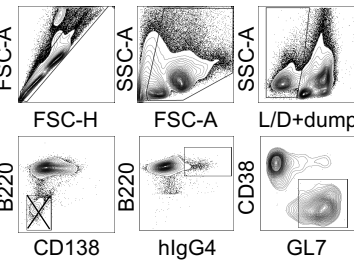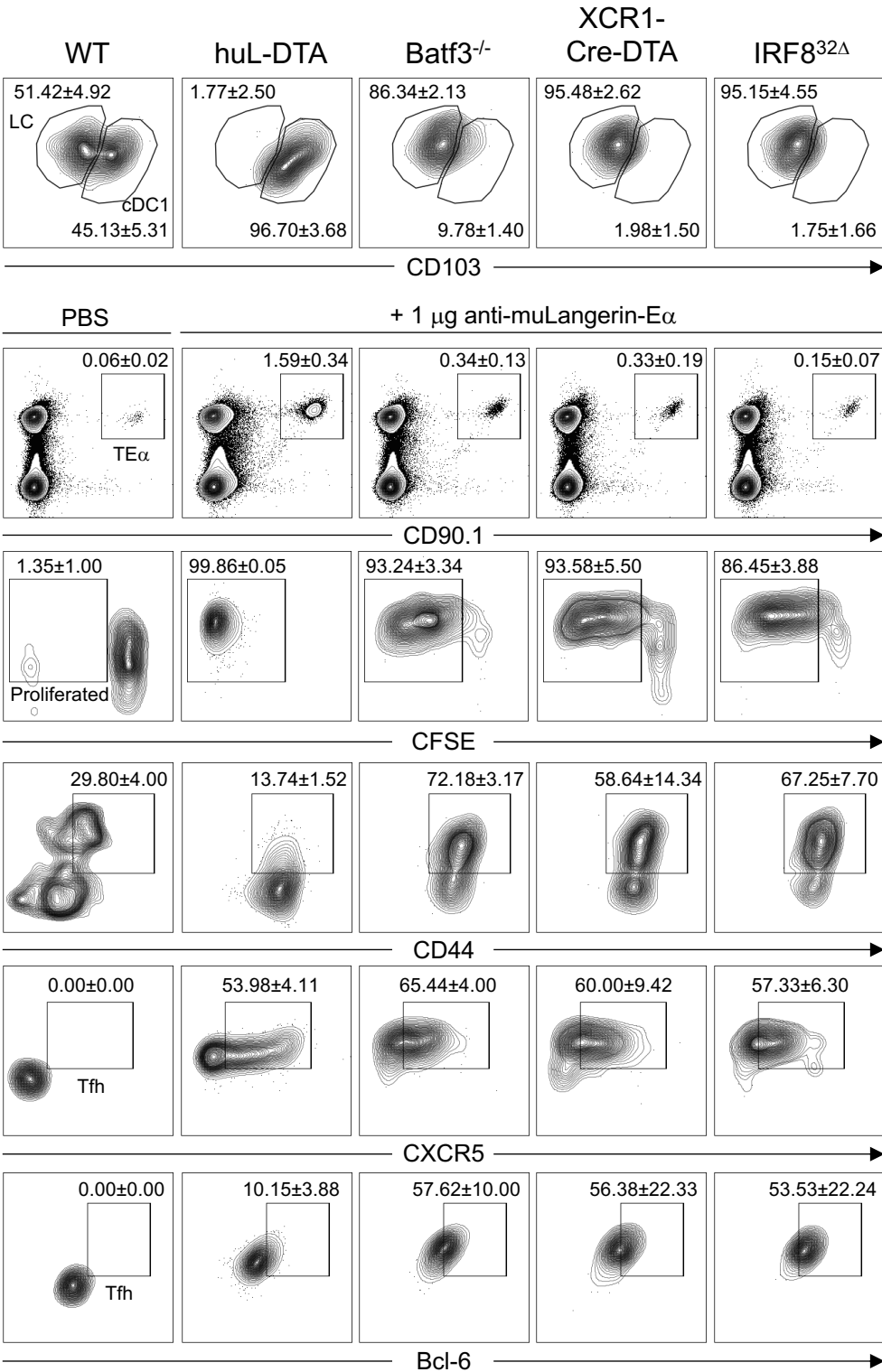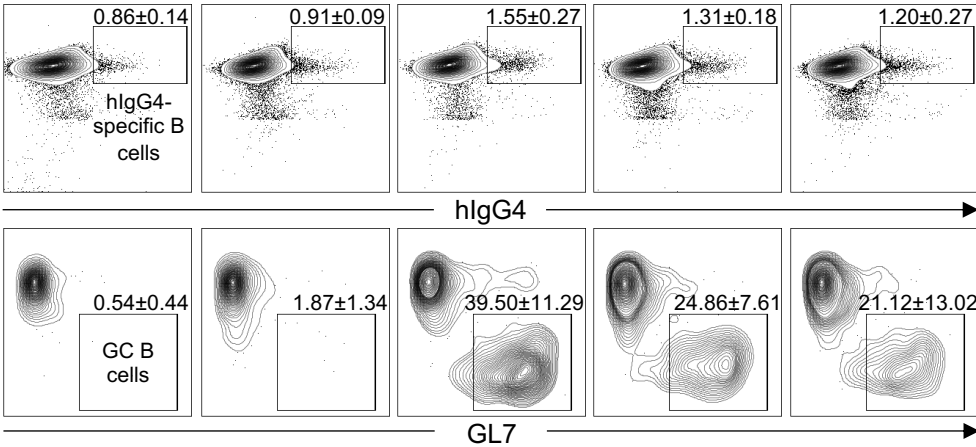

Supplement: Supplementary Figure 1 — LCs, unlike cDC1s, induce GC-Tfh cells and antibody responses in steady-state. (A) Gating strategy for LCs and cDC1s is shown on the left; representative flow cytometry plots from the indicated mouse strains are shown on the right. (B) Left: Gating strategy and phenotype of TEα cells. Right: Representative flow plots from the indicated mouse strains. (C) Left: Gating strategy and phenotype of antigen-specific B cells. Right: Representative flow plots from the indicated mouse strains. L/D = live/dead. [file Image1.pdf]

Fig. S2

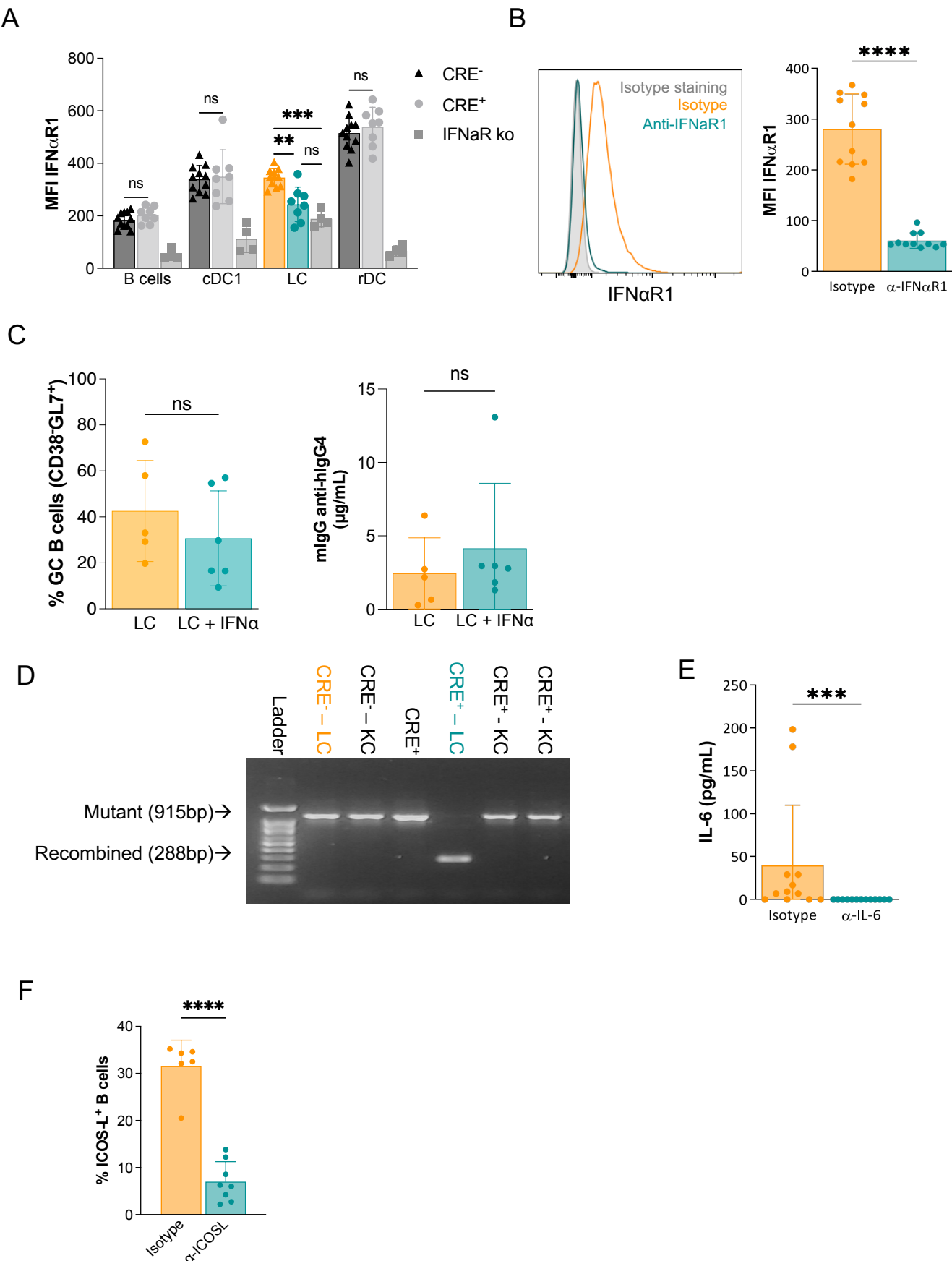

Supplement: Supplementary Figure 2 — Validation experiments for type I interferon, IL-6, and ICOS-L interference. (A) huLangCre-IFNαR1f/f mice were generated to delete IFNαR1 in LCs specifically. SDLN of huLangCre-IFNαR1f/f mice (Cre+), littermate controls (Cre-) and IFNαR1 complete knockout (KO) mice were stained for IFNαR1 by flow cytometry. MFI of IFNαR1 was calculated for B cells, cDC1s, LCs, and rDCs. Data from multiple experiments pooled together. Each dot represents a separate mouse. (B) Batf3-/- mice were treated with anti-IFNαR1 blocking Ab or an isotype. Four and fourteen days after LC targeting, SDLN were isolated and stained for IFNαR1 (same clone used in vivo to block the receptor). Left: representative histogram of B cells from isotype or anti-IFNαR1 treated mice. Shaded grey are B cells stained with an isotype control. Right: summary data of IFNαR1 staining on B cells. (C) LCs were targeted with 1 μg of anti-mLangerin-hIgG4 Ab in the absence or presence of IFNα. Fourteen days later, the percentage of GC-B cells among hIgG4-specific B cells was assessed by flow cytometry (left), and anti-hIgG4 Ab responses (right) were assessed by ELISA on serum. Data from two experiments were pooled. Each dot represents a separate mouse. (D) huLangCre-IL-6f/f mice were generated to target IL-6-deficient (LCΔIL-6) or -sufficient LCs. LCs and keratinocytes (KC) of Cre+ and Cre- mice were sorted, and genomic DNA was extracted for genotyping. Note that the recombined band is only present in Cre+ LCs. (E) The serum of Batf3-/- mice treated with anti-IL-6 or isotype control antibodies was collected, and the concentration of IL-6 was assessed by Luminex. (F) The efficiency of ICOS-L blockade is shown. B cells were stained with anti-ICOS-L. Each dot represents a separate mouse. **p<0.005, ***p<0.001, ****p<0.0001, ns=not significant. [file Image2.pdf]

Fig. S3

A

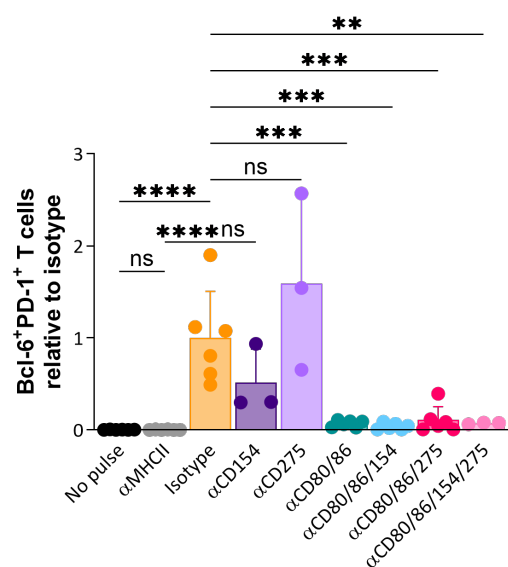

**B**

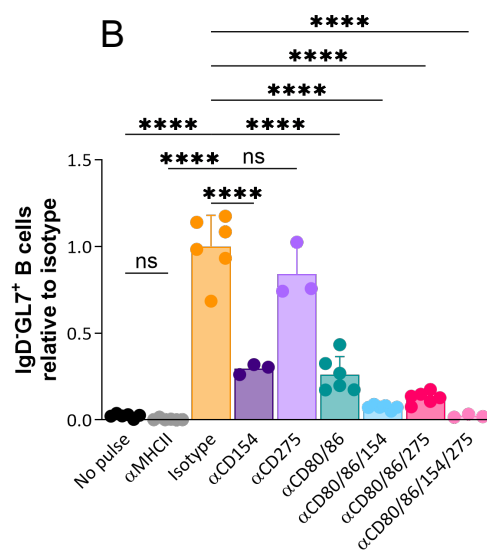

C

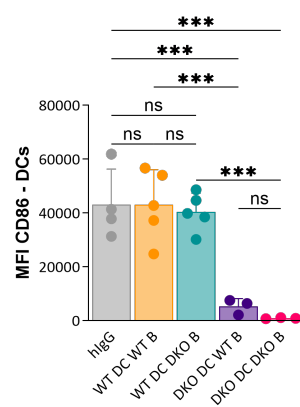

D

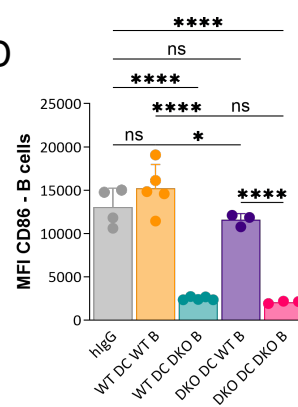

Supplement: Supplementary Figure 3 — Validation experiments for CD80/CD86 and other co-stimulatory molecules. (A) The role of MHC-II, CD154, CD275, CD80/86, or a combination of these parameters in GC responses was tested in vitro. Blocking Abs or isotype control Abs were added to the in vitro model simultaneously with T cells. Five days later, the phenotype of T cells (left) and B cells (right) was assessed by flow cytometry. The Tfh and GC B cells in each well were calculated and plotted relative to the average of Tfh and GC B cells in isotype conditions. Data from two independent experiments were pooled. Each dot represents an independent replicate. (C) At the end of the in vitro cultures with WT and CD80/86 DKO DCs and B cells, the level of CD86 on DCs and (D) B cells was determined by flow cytometry, and the MFI values of CD86 were plotted. Data from two independent experiments were pooled. [file Image3.pdf]
